# Youth E-cigarette use in the United States: carcinogenic exposure, epidemiologic trends, data gaps, and implications for cancer prevention policy

**DOI:** 10.3389/fonc.2026.1806958

**Published:** 2026-04-27

**Authors:** Balaji Kolasani, James K. Akkidas, Hemanth Rudraraju, Sravani Madamanchi

**Affiliations:** 1 Private Practitioner, Indianapolis, Indiana, United States; 2 Private Practitioner, Hershey, Pennsylvania, United States; 3 Private Practitioner, Manteca, CA, United States; 4 Independent Practitioner, Indianapolis, Indiana, United States

**Keywords:** cancer, e cigarette, lung cancer, nicotine, oral health, policy, smoking, vaping

## Introduction

Electronic cigarettes, also known as e-cigarettes, are the most common form of Electronic Nicotine Delivery Systems (ENDS), which also include vapes, vape pens, hookah pens, vaporizers, e-pipes and e-cigars. E-cigarettes heat a solution (e-liquid) to create an aerosol which frequently contains flavorants, usually dissolved into Propylene Glycol or/and Glycerin. The liquid component of e-cigarettes contains nicotine (in ENDS), propylene glycol, with or without glycerol and flavorants (“^1^, ^2^). E-cigarettes were introduced in North America in 2007 and marketed as a lower risk alternative to traditional cigarette smokers, however they are not completely risk-free. They release harmful chemicals and contain nicotine, which is highly addictive (3).

E-cigarette smoking releases a number of toxic chemicals like acetaldehyde, formaldehyde and acrolein which can cause COPD, lung cancer, pulmonary and cardiovascular diseases (4, 5). E-cigarette aerosols contain nicotine, flavors, volatile organic compounds, heavy metals, carcinogens and other hazardous chemicals which impact the oral cavity causing gingivitis, periodontitis, oral inflammation, pre-malignant oral epithelial lesions (6). Therefore, it is important to closely examine the byproducts and their interactions with biological systems, particularly the oral cavity, as it is the first point of contact.

There are limited studies on the effects of aerosol exposure on oral inflammation, DNA damage, and pathways to oral cancer (7). It is important to highlight that some e-cigarette aerosol components have been reported to have a direct impact on oral cells (6, 8). According to a study by Li Q. et al., side effects believed to be linked to artificial flavors, especially cinnamon and menthol, include altered taste perception, black tongue, hairy tongue, and nicotine stomatitis.

Many e-liquid flavors are available in the market targeting younger users but only 41 e- cigarettes were authorized by the FDA in the USA on December 20, 2025 (9).

This article highlights the different effects of e-cigarettes on oral cancer, data availability, and long-term development and implementation of policies.

## Discussion

### Safer than cigarettes

E-cigarettes can be used in a wide variety of ways. For example, an occasional smoker who transitioned to e-cigarettes or an experienced smoker who is trying to quit the smoking habit (10), or a totally non-smoker drawn towards it out of curiosity (11). Regardless, the false assumption that e-cigarettes can be a less harm than traditional cigarettes is false and lacks long term data. (12, 13). Additionally, they are designed to be sleek and easy to carry anywhere and made attractive by adding different flavors while releasing odorless vapors or smoke.

A meta-analysis conducted by Wang RJ et. al in 2021 (14) concluded that e-cigarettes in general, do not correspond to a higher smoking quit rate in the general population but are considered as a prescription drug to promote smoking cessation under clinical supervision (15). A study conducted by Mustaq N et al. using the Fagerstrom test, corroborated the addictive potential of e-cigarettes, like conventional cigarettes, through strong nicotine addiction pathways in the brain. The ENDS, along with providing nicotine, also produce harmful aerosols with cytotoxic and cellular immunomodulation effects, which can cause the development of clinical disease related to the lungs.

### Youth exposure

E-cigarettes are the most commonly used tobacco product among U.S. youth. Most e-cigarettes contain nicotine which can harm adolescent’s brain thereby affecting attention, learning, mood and impulse control (16) and is highly addictive. An article from the New England Journal of Medicine reported a 10% increase in adolescents using e-cigarettes between 2017 and 2018 alone, which is approximately 1.3 million teenagers (17). In 2024, 1.63 million (5.9%) students in the US reported using e-cigarettes which includes 410,000 (3.5%) middle school students and 1.21 million (7.8%) high school students (18, 19).

Extensive advertising and marketing by the tobacco companies, availability of a wide variety of appealing flavors, social influences and the addictive effects of nicotine contribute to the reasons for vaping among youth. The most used e-cigarette brands are Elf Bar (36.1%), Breeze (19.9%), Mr. Fog (15.8%), Vuse (13.7%) and JUUL (12.6%) (20, 21). JUUL contains approximately 5% or 59mg/ml of nicotine which is equal to the nicotine contained in 20 cigarettes (22). Despite that 39% of adolescents did not consider JUUL to be an e-cigarette at all (22) and another survey revealed that 63% of adolescents did not know that JUUL pods contained nicotine (23). E-cigarettes can also be used to inhale marijuana and a study showed that teens who use nicotine liquid in e-cigarettes were 3.6 to 4 times more likely to use marijuana in the next 2 years (24).

Parents and caregivers, educators, health care providers, states and territories, communities play a very important role in educating and bringing awareness about the harmful effects of e-cigarettes and helping them to reject or quit vaping and all other tobacco products.

### Oral health implications

The first part of the body that comes in contact with the inhaled e-aerosol is the oral cavity and is directly exposed to the chemical components and toxins. As a result, there are changes in the oral microbiome with evidence of both compositional and functional shift in the oral microbiome and increase in opportunistic pathogens (25).

E-cigarettes are known to contain formaldehyde, acetaldehyde and heavy metal constituents. Although e-cigarettes have fewer carcinogens compared to the traditional cigarettes, the mechanism of damage to DNA is similar. Few *in-vitro* studies have shown that e-cigarette vapor or liquid can induce DNA damage, oxidative stress, DNA double-stranded breaks, apoptosis, necrosis and genotoxicity in various oral cells (26–28).

One common assumption is that nicotine in e-cigarettes is the major cause for toxicity and non-nicotine e-cigarette is inert and safe. Study by Yu et al. (29) demonstrated that the vapors from e-cigarettes induced cell death through apoptosis or necrosis and caused DNA strand breaks irrespective of the presence or absence of nicotine. Although it is not clear whether the DNA strand breaks were significant enough to cause cancer, there is sufficient evidence to show that e-cigarettes with or without nicotine cannot be considered to be inert (25).

### Gaps in long-term evidence

Although e-cigarettes were linked to gum diseases, dry mouth, decay, stains, most dental professionals are not fully aware of the risks (30). Oral health professionals should be educated about the ill effects of e-cigarettes on oral health using evidence-based studies.

Many oral cancers can be diagnosed using biomarker studies. Emerging research highlights the potential of salivary biomarkers, such as microRNAs, in detecting oral health issues associated with tobacco and alternative smoking devices. Pro-Inflammatory cytokines have a known association with causing inflammation and periodontal disease. An increase in cytokine levels, such as IL-1β and TNF-α, is seen in the saliva of smokers who use e-cigarettes. (30).

Clinical studies require long follow up periods to evaluate the long term effects of e-cigarettes and pose ethical concerns hence early interventions should be applied by the oral health professionals. More studies should be conducted to identify early biomarker indicators in e-cigarette smokers to facilitate better care for the patients.

### Implications for prevention

As per the Tobacco 21 rule passed in 2019, people under 21 years are not allowed to buy e-cigarettes and other tobacco products. (31). However, this policy should be implemented strictly. Flavors should be limited as some flavors like diacetyl are metabolized differently in lungs compared to gut (32). Another important development is the PACT agreement, which prevents the shipping of vapes with USPS (7). The PACT agreements should be extended to other mailing services and should be sold only in person with an ID check, like any alcohol and tobacco products that are sold in stores.

### Implications for policy

E- cigarette unit sales between February 2020 and June 2024 increased from 15.7 million units to 21.1 million units (34.7% increase). In June 2024, e-cigarette sales totaled $488.9 millions. (33).

“According to a study conducted in Kosovo in Europe, a large majority of both general dentists (77.7%) and specialists (69.8%) were unsure whether e-cigarettes are FDA-approved for safe consumption, while only 2.9% and 4.7%, respectively, affirmed FDA approval. (30). We would recommend the following policy adaptations.

- All oral health professionals, including dentists and dental hygienists, must have mandatory continuing education hours on Tobacco and e-cigarettes for their license renewal from the state and national dental associations. This allows oral health professionals to keep themselves updated with the current literature in this regard.
- Inclusion of e-cigarette usage in social history questionnaires should be made mandatory during medical and dental office visits.
- School children and young adults must be educated on the ill effects of e-cigarettes, as the usage is more prevalent in these age groups. Awareness and counselling should be provided in schools, colleges, hospitals and community centers.
- Strict policies should be developed and adhered by agencies like the Food and Drug Administration (FDA), Centers for Disease Control and Prevention (CDC), the National Institutes of Health (NIH), and the Substance Abuse and Mental Health Services Administration.

## Conclusion

Despite the wide belief that E-cigarettes are a lower-risk alternative than traditional cigarettes more evidence is needed to support this statement. Further research studying patients with only e-cigarette use and no prior history of other oral cancer risk factors is needed to assess e-cigarettes as an independent risk factor for oral cancer.

The development of interdisciplinary treatment protocols involving oral health professionals, primary care providers, nurses and oncologists is crucial to improve comprehensive care for the people using e-cigarettes.

Oral health care professionals and health care providers have the responsibility to promote awareness about the toxic effects of e-cigarettes across schools, colleges, hospitals and provide resources that will help people with e-cigarette cessation.

## References

[1] Electronic Nicotine Delivery Systems and Electronic Non-Nicotine Delivery Systems (ENDS/ENNDS) . (2017). Available online at: https://www.who.int/news/item/25-01-2017-electronic-nicotine-delivery-systems-and-electronic-non-nicotine-delivery-systems-(ends-ennds) (Accessed March 15, 2026). Www.who.int.

[2] Dominika C Kusiak A Goniewicz ML . The Impact of E-cigarettes on oral health—A narrative review. Dentist J. (2024) 12:404–4. doi: 10.3390/dj12120404, PMID: 39727461 PMC11675017

[3] FDA . E-Cigarettes, Vapes, and Other Electronic Nicotine Delivery Systems (ENDS). Silver Spring, MD, USA: U.S. Food and Drug Administration (2024). Available online at: https://www.fda.gov/tobacco-products/products-ingredients-components/e-cigarettes-vapes-and-other-electronic-nicotine-delivery-systems-ends (Accessed March 15, 2026).

[4] American Lung Association . “The Impact of E-Cigarettes on the Lung.” Www.lung.org. American Lung Association (2023). Available online at: https://www.lung.org/quit-smoking/e-cigarettes-vaping/impact-of-e-cigarettes-on-lung (Accessed March 15, 2026).

[5] Szumilas P Wilk A Szumilas K Karakiewicz B . The effects of E-cigarette aerosol on oral cavity cells and tissues: A narrative review. Toxics. (2022) 10:74. doi: 10.3390/toxics10020074, PMID: 35202260 PMC8878056

[6] Ganapathy V Jaganathan R Chinnaiyan M Chengizkhan G Sadhasivam B Manyanga J . E-cigarette effects on oral health: A molecular perspective. Food Chem Toxicol. (2024) 196:115216–6. doi: 10.1016/j.fct.2024.115216, PMID: 39736445 PMC11976636

[7] www.atf.gov . *Vapes and E-Cigarettes* | *Bureau of Alcohol, Tobacco, Firearms and Explosives* (2025). Available online at: https://www.atf.gov/alcohol-tobacco/vapes-and-e-cigarettes (Accessed February 7, 2026).

[8] Li Q Zhan Y Wang L Leischow SJ Zeng DD . Analysis of symptoms and their potential associations with e-liquids’ components: a social media study. BMC Public Health. (2016) 16. doi: 10.1186/s12889-016-3326-0, PMID: 27475060 PMC4967297

[9] Center . E-Cigarettes Authorized by the FDA. Silverspring, Maryland, USA: U.S. Food and Drug Administration (2025). Available online at: https://www.fda.gov/tobacco-products/market-and-distribute-tobacco-product/e-cigarettes-vapes-and-other-electronic-nicotine-delivery-systems-ends-authorized-fda.

[10] Goniewicz ML Lingas EO Hajek P . Patterns of electronic cigarette use and user beliefs about their safety and benefits: An Internet survey. Drug Alcohol Rev. (2012) 32:pp.133–140. doi: 10.1111/j.1465-3362.2012.00512.x, PMID: 22994631 PMC3530631

[11] Weaver M Breland A Spindle T Eissenberg T . Electronic Cigarettes: a review of safety and clinical issues. J Addict Med. (2014) 8:234–40. doi: 10.1097/adm.0000000000000043, PMID: 25089953 PMC4123220

[12] Giovacchini CX Crotty Alexander LE Que LG . Electronic cigarettes: A pro–con review of the current literature. J Allergy Clin Immunol: In Pract. (2022) 10(11):2843–51. doi: 10.1016/j.jaip.2022.07.009, PMID: 35872217 PMC12922728

[13] Smith L Brar K Srinivasan K Enja M Lippmann S . E-cigarettes: how “safe” are they? J Fam Pract. (2016) 65:380–5. 27474819

[14] Wang RJ Bhadriraju S Glantz SA . E-cigarette use and adult cigarette smoking cessation: A meta-analysis. Am J Public Health. (2020) 111:e1–e17. doi: 10.2105/ajph.2020.305999, PMID: 33351653 PMC7811087

[15] Mushtaq N Beebe LA . Psychometric properties of fagerström test for nicotine dependence for smokeless tobacco users (FTND-ST). Nicotine Tobacco Res. (2017) 19:pp.1095–1101. doi: 10.1093/ntr/ntx076, PMID: 28387864

[16] CDCTobaccoFree . 2016 SGR: E-Cigarette Use Among Youth and Young Adults. Atlanta, Georgia, USA: Centers for Disease Control and Prevention (2017). Available online at: https://archive.cdc.gov/www_cdc_gov/tobacco/sgr/e-cigarettes/index.htm.

[17] Farzal Z Perry MF Yarbrough WG Kimple AJ . The adolescent vaping epidemic in the United States—How it happened and where we go from here. JAMA Otolaryngology–Head Neck Surg. (2019) 145(10):885–6. doi: 10.1001/jamaoto.2019.2410, PMID: 31436792

[18] Jamal A Lee EP Birdsey J West A Cornelius M Cooper MR . Tobacco product use among middle and high school students — National youth tobacco survey, United States 2024. MMWR Morbid Mortality Weekly Rep. (2024) 73(41):917–24. doi: 10.15585/mmwr.mm7341a2, PMID: 39418216 PMC11486349

[19] Park-Lee E Jamal A Cowan H Sawdey MD Cooper MR Birdsey J . Notes from the field: E-cigarette and nicotine pouch use among middle and high school students — United states 2024. MMWR Morbid Mortality Weekly Rep. (2024) 73:774–8. doi: 10.15585/mmwr.mm7335a3, PMID: 39236021 PMC11376506

[20] U.S. Food and Drug Administration . Results from the annual National Youth Tobacco Survey (NYTS) (2025). Available online at: https://www.fda.gov/tobacco-products/youth-and-tobacco/results-annual-national-youth-tobacco-survey-nyts (Accessed February 8, 2026).

[21] Park-Lee E . Notes from the field: E-cigarette use among middle and high school students — National youth tobacco survey, United States 2021. MMWR Morbid Mortality Weekly Rep. (2021) 70:1387–9. doi: 10.15585/mmwr.mm7039a4, PMID: 34591834 PMC8486384

[22] Morean ME Bold KW Kong G Gueorguieva R Camenga DR Simon P . Adolescents’ awareness of the nicotine strength and e-cigarette status of JUUL e-cigarettes. Drug Alcohol Depend. (2019) 204:107512. doi: 10.1016/j.drugalcdep.2019.05.032, PMID: 31487572 PMC6878179

[23] Fadus MC Smith TT Squeglia LM . The rise of e-cigarettes, pod mod devices, and JUUL among youth: Factors influencing use, health implications, and downstream effects. Drug Alcohol Depend. (2019) 201:85–93. doi: 10.1016/j.drugalcdep.2019.04.011, PMID: 31200279 PMC7183384

[24] Selekman J . Vaping: It’s All a Smokescreen - ProQuest (2019). Available online at: www.proquest.comhttps://www.proquest.com/docview/2184907265?sourcetype=Scholarly%20Journals (Accessed February 7, 2026).

[25] Cameron A Yip HM Garg M . Current thinking about the effects of e-cigarettes on oral cancer risk. Br Dental J. (2024) 236:397–400. doi: 10.1038/s41415-024-7124-2, PMID: 38459320

[26] Wilson C Tellez Freitas CM Awan KH Ajdaharian J Geiler J Thirucenthilvelan P . Adverse effects of E-cigarettes on head, neck, and oral cells: A systematic review. J Oral Pathol Med. (2022) 51:113–25. doi: 10.1111/jop.13273, PMID: 35048431

[27] Yang I Sandeep S Rodriguez J . The oral health impact of electronic cigarette use: a systematic review. Crit Rev Toxicol. (2020) 50:1–30. doi: 10.1080/10408444.2020.1713726, PMID: 32043402

[28] Raj AT Sujatha G Muruganandhan J Kumar SS Bharkavi SI Varadarajan S . Reviewing the oral carcinogenic potential of E-cigarettes using the Bradford Hill criteria of causation. Trans Cancer Res. (2020) 9:pp.3142–3152. doi: 10.21037/tcr.2020.01.23, PMID: 35117678 PMC8798817

[29] Yu V Rahimy M Korrapati A Xuan Y Zou AE Krishnan AR . Electronic cigarettes induce DNA strand breaks and cell death independently of nicotine in cell lines. Oral Oncol. (2016) 52:58–65. doi: 10.1016/j.oraloncology.2015.10.018, PMID: 26547127 PMC4891196

[30] Shabani DB Dula LJ Dalipi ZS Krasniqi MS Meto A . Knowledge and perceptions of dentists regarding E-cigarettes: implications for oral health and public awareness and education. Dentist J. (2025) 13:119. doi: 10.3390/dj13030119, PMID: 40136747 PMC11941090

[31] Tobacco . Tobacco 21. Indianapolis, IN, USA: Tobacco Prevention & Cessation (2021). Available online at: https://www.in.gov/health/tpc/tobacco-21/.

[32] CDC . Health effects of vaping. In: Smoking and Tobacco Use. Atlanta, Georgia, USA: (CDC headquarters) Centers for Disease Control and Prevention (CDC) (2025). Available online at: https://www.cdc.gov/tobacco/e-cigarettes/health-effects.html.

[33] CDC . About E-Cigarettes (Vapes). In: Smoking and Tobacco Use. Atlanta, Georgia, USA: (CDC headquarters) Centers for Disease Control and Prevention (CDC). (2024). Available online at: https://www.cdc.gov/tobacco/e-cigarettes/about.html.

